# RHAPSODY, Biomarkers and Novel Clinical Trial design in type 2 diabetes (T2D) and prediabetes

**DOI:** 10.1002/edm2.207

**Published:** 2020-12-06

**Authors:** Rachel Adams, Nicole Daly, Elizabeth Robertson, Giuseppe N. Giordano

**Affiliations:** ^1^ Janssen High Wycombe Bucks UK; ^2^ Diabetes UK Wells Lawrence House London UK; ^3^ Department of Clinical Sciences Genetic and Molecular Epidemiology Unit, LUDC Lund University Skåne University Hospital Malmö Sweden

**Keywords:** biomarkers, disease progression, novel clinical trial design, prediabetes, type 2 diabetes mellitus

## Abstract

Developing a novel therapeutic product for the treatment of type 2 diabetes (T2D) is a long, resource‐intensive process. Novel biomarkers could potentially aid clinical trial design by shortening clinical trials or enabling better prediction of at‐risk populations and/or disease progression. Novel clinical trial designs could lead to reduced costs of development and less burden to patients, due to shorter trial duration, and/or less burdensome assessments.

## Graphical abstract

This is a review of the potential use of biomarkers in novel clinical trial design. It also describes the IMI2 RHAPSODY consortium.

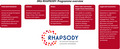

Developing a novel therapeutic product for the treatment of T2D is a long, resource‐intensive process. However, it is important that its risks and benefits are evaluated in the context of relevant populations and conditions of use, so that adequate prescribing information can be provided to physicians, healthcare professionals and patients.

RHAPSODY is an IMI2 public‐private consortium (https://imi‐rhapsody.eu/project). Its aim is to define a molecular taxonomy of T2D that could support patient segmentation, inform clinical trial design, and the establishment of regulatory paths for the adoption of novel strategies for diabetes prevention and treatment.

RHAPSODY plans are built upon:

- access to large European cohorts with comprehensive genetic analyses, rich longitudinal clinical, biochemical data and biomarker samples
- detailed multiomic maps of key T2D‐relevant tissues and organs
- extensive expertise in the development and use of novel genetic, epigenetic, biochemical and physiological experimental approaches
- the ability to combine existing and novel data sets, through effective data federation, and to use these in systems biology approaches towards precision medicine;
- expertise in regulatory approval, health economics and patient engagement.

The work programme within RHAPSODY aims to uncover novel biomarkers that could potentially aid clinical trial design by shortening clinical trials or enabling better prediction of at‐risk populations and/or disease progression. Novel clinical trial designs could lead to reduced costs of development and less burden to patients, due to shorter trial duration, and/or less burdensome assessments. Biomarkers of disease progression could lead to greater understanding of the course of the disease or specific symptoms of the disease. Biomarkers that identify particular patient groups may allow for more specific targeting of therapy. If the heterogeneity of treatment effect, that is observed in broad‐based population studies, could be overcome through using biomarkers to more precisely identify high‐risk subjects at baseline more likely to respond to a specific intervention, this could lead to smaller trials and would also be a step towards precision medicine implementation. The technological advances that have driven the omics revolution also hold the potential to provide alternative approaches to clinical trial design, both for T2D and prediabetes. In addition, current evaluation of therapeutic effectiveness remains primarily focused on the measurement of glycated haemoglobin (HbA1c). Though a well‐recognized end‐point of long‐term blood glucose control, HbA1c provides limited information on other important end‐points of this multifaceted disease and may not predict the full burden of disease on the patient.

## 1 STRATIFICATION OF PATIENT POPULATION BASED ON BIOMARKERS

Currently, there are few data sets available on the prospective use of genetic and/or biomarkers for patient selection in clinical trials for T2D. As each patient is unique with different personal goals, medical history and expectations, only a general evaluation of the efficacy of a therapeutic agent over a range of types of patients can be made. It would be extremely useful to be able to select patients based upon potential biomarkers for disease progression or disease status. Biomarkers identified within RHAPSODY could potentially allow for such selection.

Enrolling clinical trial populations based on biomarker profiles may facilitate improved clinical trial efficiency and conduct.^1^ Novel prognostic biomarkers could be used to enrich clinical trial populations, selecting patients at high risk of developing specific events of interest (e.g. cardiovascular or renal events). This can reduce clinical trial sample sizes. Examples of such population enrichment in diabetic kidney disease (DKD) trials have been performed with the biomarkers tumour necrosis factor‐1 and kidney injury molecule‐1.^2^ In addition, the PRIORITY trial uses a panel of biomarkers consisting of 273 peptides to identify individuals at high risk of developing microalbuminuria.^3^

Biomarkers can also be used to target populations that are more likely to respond.^1^ An example of such a predictive biomarker is reported in a study^4^ which demonstrates that polymorphisms in the ACE gene predict the response to angiotensin receptor blockers in patients with diabetic kidney disease. The use of novel predictive biomarkers could, therefore, provide opportunities to predict treatment outcomes more accurately, ultimately leading to smaller and more efficient trials. These biomarkers could be genes, proteins, lipids or metabolites identified in RHAPSODY.

## 2 MONITORING DISEASE PROGRESSION BASED ON BIOMARKERS

Clinical trials use end‐points of disease progression to evaluate efficacy and safety. Sometimes these end‐points take many years to develop. Having a biomarker that is a surrogate for disease end‐points could potentially save time in determining if a treatment is effective.

A biomarker of disease progression would allow assessment of specific disease outcomes. For example, cardiovascular outcome trials are large and run for many years. However, the size (10 000s patients) and length of clinical trials (5 years +) needed to evaluate therapies using observational methods are prohibitive to the development of therapeutics. For novel therapeutic agents for T2D, the evaluation of any effects of new agents on cardiovascular outcomes is also required by regulators. If there were biomarkers of cardiovascular disease progression within prediabetes and T2D, this could mean improved CV safety and reduced cost, with shorter, smaller clinical trials. Similarly for therapies developed for the treatment of diabetic kidney disease, currently regulators mandate the need for ‘hard outcome’ event‐driven studies, which are consequently extensive in terms of study duration and cost. The identification of novel biomarkers for use with other clinically meaningful surrogate end‐points could allow for a more rapid efficacy assessment in the future. However, shortening the timeframe of a therapeutic trial based solely upon biomarker behaviour may reduce opportunities to observe unintended, longer‐term, off‐target effects.

## 3 INCORPORATING PATIENT‐REPORTED OUTCOMES, DIGITAL TECHNOLOGY AND REAL‐WORD EVIDENCE

The impact of a therapeutic, as experienced by the patient, is an important consideration. Regulators are increasingly involving patients in the review of new medicines, asking them to provide input on which measures are clinically meaningful to their daily lives, and their perception of benefits and risks. Furthermore, payers are requiring evidence that medicines are adding value to the patient's quality of life.

Patients are also being involved in many aspects of drug development, including aiding in the design of trials (*co‐design*). Though this is in its infancy, greater efforts are being made to maximize patient involvement in this area by industry.^5^

The use of continuous glucose‐monitoring (CGM) technologies to monitor time‐in‐range and patient‐reported outcome measures (PROMs) could also be utilized to assess impact on sleep patterns, anxiety, diabetes distress or resilience. A stepped or adaptive trial design approach, use of real‐world evidence, actively recruiting ethnic minorities and limiting the (usual) exclusion of comorbid patients would further help with future study‐population stratification, while creating a more holistic understanding of the efficacy of the trials and their impact on patients.

Table 1 below describes potential opportunities, strengths, limitations and caveats of biomarkers in particular reference to RHAPSODY. It is envisaged that biomarkers identified in the RHAPSODY programme would initially be used in a clinical trial setting, potentially for either stratifying patient selection or monitoring disease progression.

**Table 1:** Strengths, limitations, opportunities and caveats related to RHAPSODY novel biomarker use

**Strengths** Prognostic and diagnostic utilization Target individuals more likely to respond to interventions Predict treatment outcomes more accurately and earlier May lead to greater understanding of disease progression	**Limitations** Thorough validation and replication required including validity, accuracy, variability, reliability, interpretability and feasibility Biomarker must accurately predict clinical outcome Cost and ease of detection vs. current, cheap gluco‐centric tests in both clinical trial and clinical practice settings. Translation into clinical practice
**Opportunities** Overcomes limitations of current gluco‐centric models of diagnosis and treatment Enable stratification of population, based upon biomarkers for disease progression or status Improve clinical trial efficiency, shorten length and conduct Improvement in public health	**Caveats** Acceptance from professionals and public Shorter trials, assessed solely on the behaviour of surrogate end‐points, may reduce opportunities to observe longer‐term, off‐target effects. High cost

## Data Availability

Data sharing is not applicable to this article as no new data were created or analysed in this study.
